# A The complete chloroplast genome and phylogenetic analysis of Bupleurum yinchowense Shan & Yin Li

**DOI:** 10.1080/23802359.2020.1866465

**Published:** 2021-03-30

**Authors:** Gaixia Zhang, Weijun Kong, Qiuling Wang, Fuhua Lu, Yue Jin, Jiemei Jiang, Linchun Shi

**Affiliations:** Institute of Medicinal Plant Development, Chinese Academy of Medical Sciences & Peking Union Medical College, Beijing, China

**Keywords:** Bupleurum, *Bupleurum yinchowense*, chloroplast genome, phylogenetic analysis

## Abstract

*Bupleurum yinchowense* Shan & Yin Li was first described as a new *Bupleurum* species in 1974, but its classification status has always been disputed. Here, its complete chloroplast genome was provided to resolve this issue. The length of the *B. yinchowense* chloroplast genome is 155,851 bp and composed of two inverted repeats (IR: 26,307 bp), a large single-copy region (LSC: 85,625 bp), and a small single-copy region (SSC: 17,612 bp). The overall GC content is 37.6%. The chloroplast genome consists of 113 genes, including 79 protein-coding genes, four rRNA genes, and 30 tRNA genes. Phylogenetic analysis suggested that *Bupleurum yinchowense* holds a distinct phylogenetic position and can be considered as an accepted species.

*Bupleurum yinchowense* Shan & Yin Li is a perennial plant in the *Bupleurum* genus of the Apiaceae family (Wu et al. 2005) and was first recognized as a new species in 1974 by Shan and Li (1974). *Bupleurum yinchowense* is 20–50 cm high with a stout, long root and is widely used as a substitute for the Chinese herbal medicinal material Bupleuri Radix (Chai hu) (Wu et al. 2005), *B. yinchowense* is commonly recorded as ‘Yinzhou-Chai hu’ and is considered having the best quality medicinal properties (Wang et al. 1992). However, Liu and Shang (1994) believed that the source of ‘Chai hu’ in ancient ‘yinzhou’ is another species of *Bupleurum* named *B. scorzonerifolium* Willd. In addition, the morphological characteristics of *B. yinchowense* were extraordinarily similar to those of *B. angustissimum* (Franch.) Kitag. and *B. bicaule* Helm (Shan and Li 1974; Wu et al. 2005). Though chloroplast genomes have highly conserved properties with respect to genome size, organization, and gene content, they also contain a diversity of genetic polymorphisms, which means they are an ideal research tool for resolving the phylogenetic relationships of previous recalcitrant nodes (Asaf et al. 2017; Jiang et al. 2017). Here, in order to evaluate the genetic information and taxonomic position more accurate and reliable, the complete chloroplast genome of *B. yinchowense* has been sequenced based on high-throughput sequencing technology.

Fresh leaf samples of *B. yinchowense* were collected from Moli Town (Jiangxian County, Yuncheng City, Shanxi Province N35° 30'48.59’, E111° 43'36.99’). A voucher specimen was stored in the herbarium of the Institute of Medicinal Plant Development, the Chinese Academy of Medical Sciences and Peking Union Medical College. The herbarium code was ‘IMD’ (NYBG: https://www.nybg.org/). Total genomic DNA was extracted from the leaf materials of one *B. yinchowense* specimen (sample ID SXW02-1; voucher number HPAB0001) using the modified CTAB-based extraction method described by Porebski et al. (1997). The DNA quality and concentration were measured using a NanoDrop ONE ultra-micro spectrophotometer (Thermo Fisher Scientific Inc., USA). An Illumina PCR-free library (∼350 bp) was constructed and sequenced using the Illumina NovaSeq platform, generating 8,140,617 paired-end reads, totaling 2.44 Gb. The sequencing adapter and low-quality reads were filtered using Trimmonmatic v0.38 (Bolger 2014). The complete chloroplast genome of *B. yinchowense* was assembled using a NOVOPlasty Toolkit (Dierckxsens et al. 2016). Furthermore, the genome sequence was independently annotated using the CPGAVAS2 webserver (Shi et al. 2019). The chloroplast genome of *B. yinchowense* has been submitted to GenBank and its accession number is MT075711.

The chloroplast genome of *B. yinchowense* was circular, and its size was 155,851 bp. Two IR regions were evident (26,307 bp), showing one LSC region (85,625 bp) and one SSC region (17,612 bp). The *B. yinchowense* chloroplast genome displayed a G/C content of 37.6%, while it included a total of 113 genes, of which 79 were protein-coding genes, four were rRNA genes, and 30 were tRNA genes.

Furthermore, 38 chloroplast genomes of 37 species, including 35 species in the Apiaceae family, one species (*Panax ginseng*) in the Araliaceae family, and one species (*Cornus capitata*) in the Cornaceae family, were used for phylogenetic analysis. The maximum likelihood (ML) tree was constructed using RAxML *v*8.0.0 (Stamatakis 2014) with 1000 bootstrap replicates, while the *Panax ginseng* and *Cornus capitata* species were used as the outgroups (Figure 1). The phylogenetic tree showed that *B. yinchowense* and other *Bupleurum* species could form a monophyletic group. In particular, the phylogenetic tree indicated that *B. yinchowense* is an accepted species and has a close relationship with the *B. scorzonerifolium* species. The complete chloroplast genome of *B. yinchowense* provided substantial genetic information for the phylogenetic analysis of the *Bupleurum* genus. In addition, the results regarding the complete chloroplast genome of *B. yinchowense* imply that the root can be used as a new source for the herbal Chinese medicinal material, Bupleuri Radix, especially considering its chemical data (Fu et al. 2011).

**Figure 1.**
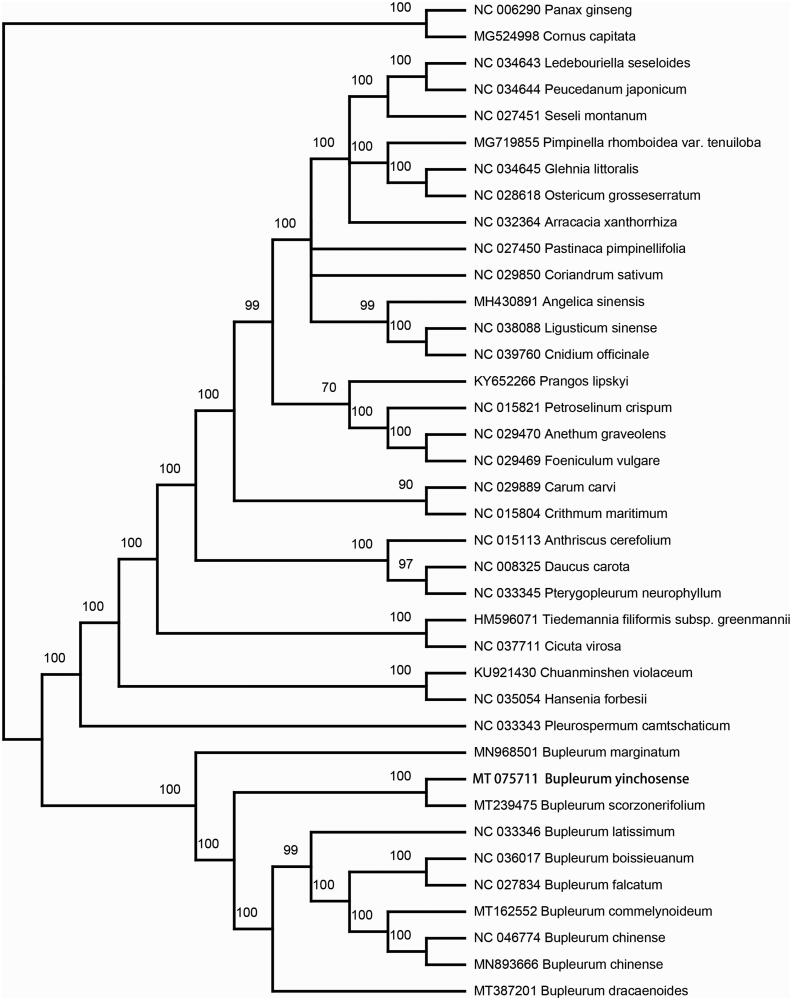
The phylogenetic position for *B. yinchowense* according to the ML phylogenetic tree constructed based on 38 chloroplast genomes. The bootstrap support values are shown on the nodes.

## Data Availability

The genome sequence data that support the findings of this study are openly available in GenBank of NCBI at (https://www.ncbi.nlm.nih.gov/) under the accession no. MT075711. The associated BioProject, SRA, and Bio-Sample numbers are PRJNA682316, SRR13189613, and SAMN16987560 respectively.
